# HSV-2 Acute Retinal Necrosis Complicated by Persistent Vitreous Opacities and Cystoid Macular Edema: A Case Report

**DOI:** 10.51894/001c.164426

**Published:** 2026-08-01

**Authors:** Abhinav Ankem, Katherine Jaje, Farhan Hussain

**Affiliations:** 1 College of Osteopathic Medicine, Michigan State University

## 60

### INTRODUCTION

Acute retinal necrosis (ARN) is a rare, vision-threatening necrotizing herpetic retinopathy characterized by panuveitis, peripheral retinal necrosis, occlusive vasculopathy, and a high risk of retinal detachment. Although herpes simplex virus type 2 (HSV-2) is a relatively uncommon cause of ARN in adults, prompt diagnosis and antiviral therapy are crucial in preserving vision. Despite appropriate management, chronic inflammation and structural complications may still limit visual recovery.

### CASE DESCRIPTION

A 44-year-old man with a history of neonatal HSV-2 infection, recurrent cutaneous herpes, prior varicella, and thoracic shingles presented with left eye pain, redness, and an inferotemporal scotoma. Ophthalmologic examination revealed 4+ anterior chamber cells, vitreous cells, mild optic disc edema, and superonasal peripheral retinal whitening. Fluorescein angiography showed ischemia over the lesions with optic nerve hyperfluorescence. Anterior chamber tap PCR was positive for HSV-2 and negative for cytomegalovirus, varicella-zoster virus, and Toxoplasma gondii, confirming HSV-2 ARN. He received treatment with intravitreal foscarnet and ganciclovir, intravitreal acyclovir followed by a prolonged course of oral valacyclovir, and topical and oral corticosteroids. After tapering and discontinuation of therapy, his vision was limited due to vitreous debris; thus, he underwent pars plana vitrectomy with endolaser and membrane peel 8 months following his initial ARN diagnosis. Post-operatively, his retina remained attached without signs of recurrent infection; however, he developed elevated intraocular pressure and a cataract requiring cataract extraction and intraocular lens placement. He later required sub-Tenon’s triamcinolone due to pseudophakic cystoid macular edema.

### CONCLUSION

This case illustrates the aggressive course and complications of HSV-2-associated ARN despite appropriate therapy. Continuous antiviral suppression and patient adherence are critical in preventing recurrence and maintaining disease control. Persistent vitreous opacities and cystoid macular edema may restrict visual recovery even after infection resolution. Notably, this case underscores for clinicians and trainees the importance of long-term follow-up in HSV-related retinopathies to detect and treat vision-threatening complications early.

